# Isolation and characterization of a small antiretroviral molecule affecting HIV-1 capsid morphology

**DOI:** 10.1186/1742-4690-6-34

**Published:** 2009-04-08

**Authors:** Samir Abdurahman, Ákos Végvári, Michael Levi, Stefan Höglund, Marita Högberg, Weimin Tong, Ivan Romero, Jan Balzarini, Anders Vahlne

**Affiliations:** 1 Division of Clinical Microbiology, Karolinska Institutet, F68 Karolinska University Hospital Huddinge, SE-141 86 Stockholm, Sweden; 2 Tripep AB, Hälsovägen 7, SE-141 57 Huddinge, Sweden; 3 Department of Electrical Measurements, Lund University, SE-221 00 Lund, Sweden; 4 Department of Biochemistry, Uppsala University, SE-751 23 Uppsala, Sweden; 5 Chemilia AB, SE-141 83 Huddinge, Sweden; 6 Rega Institute for Medical Research, Katholieke Universiteit Leuven, Minderbroedersstraat 10, B-3000 Leuven, Belgium

## Abstract

**Background:**

Formation of an HIV-1 particle with a conical core structure is a prerequisite for the subsequent infectivity of the virus particle. We have previously described that glycineamide (G-NH_2_) when added to the culture medium of infected cells induces non-infectious HIV-1 particles with aberrant core structures.

**Results:**

Here we demonstrate that it is not G-NH_2 _itself but a metabolite thereof that displays antiviral activity. We show that conversion of G-NH_2 _to its antiviral metabolite is catalyzed by an enzyme present in bovine and porcine but surprisingly not in human serum. Structure determination by NMR suggested that the active G-NH_2 _metabolite was α-hydroxy-glycineamide (α-HGA). Chemically synthesized α-HGA inhibited HIV-1 replication to the same degree as G-NH_2_, unlike a number of other synthesized analogues of G-NH_2 _which had no effect on HIV-1 replication. Comparisons by capillary electrophoresis and HPLC of the metabolite with the chemically synthesized α-HGA further confirmed that the antiviral G-NH_2_-metabolite indeed was α-HGA.

**Conclusion:**

α-HGA has an unusually simple structure and a novel mechanism of antiviral action. Thus, α-HGA could be a lead for new antiviral substances belonging to a new class of anti-HIV drugs, i.e. capsid assembly inhibitors.

## Background

During or concomitant with the HIV-1 release from infected cells, the Gag precursor protein (p55) is cleaved sequentially into matrix (MA/p17), capsid (CA/p24), nucleocapsid (NC/p7) and p6. Thus, proteolytic cleavage of p55 within the budded particle triggers a morphological change of the core which transforms it from a spherical [1] to a conical core structure consisting of approximately 1,500 p24 molecules [2-4]. The conical core formation not only results as a consequence of the proteolytic cleavage of p55 but also from substantial conformational changes and rearrangements of the p24 [1] which is connected to one another through N-terminal hexamer and C-terminal dimer formations [5-8]. Acquisition of virion infectivity, reverse transcription, and subsequent dissociation of the capsid core are all critically dependent on just the right semi-stability of the capsid cone structure, which in turn is made up of multiple semi-stable non-covalent p24-p24 interactions [9]. Thus, proper structural rearrangement of p24 after Gag cleavage is a crucial step and is a highly conserved feature in most retroviruses [10]. This makes p24 of interest as a target for developing new antiviral drugs.

There are twenty-five approved drugs that belong to six different antiretroviral classes for the treatment of HIV-patients [11]. The majority of these drugs control HIV-1 infection by targeting the two viral enzymes reverse transcriptase and protease [12]. A 36 amino acid peptide binding to the transmembrane glycoprotein gp41 inhibiting the fusion of the viral envelope with the plasma membrane is also used [13,14]. Two other classes of antiretroviral drugs, a CCR5 co-receptor antagonist entry inhibitor [15] and an integrase inhibitor [16,17], have also recently been approved. Other drugs being developed include zinc finger inhibitors affecting the RNA binding of the nucleocapsid protein (NC, p7) [18,19], and capsid maturation inhibitors [20-22].

We have previously shown that the tripeptide glycyl-prolyl-glycineamide (GPG-NH_2_) cleaved to G-NH_2 _by dipeptidyl peptidase CD26, present in both human and fetal calf serum, affects proper HIV-1 capsid assembly and infectivity [23-26]. Here we show that G-NH_2 _by itself does not affect HIV-1 replication, but displays antiviral effect only when converted to a metabolite by a yet uncharacterized enzyme present in porcine or bovine serum but not in human serum. The metabolite was identified as the small molecule α-hydroxy-glycineamide (α-HGA) having a molecular mass of only 90 Daltons, a molecule which we recently showed could inhibit HIV-1 replication [27].

## Results

### The effect of serum on the antiviral activity of glycineamide (G-NH_2_)

**Figure 1:**
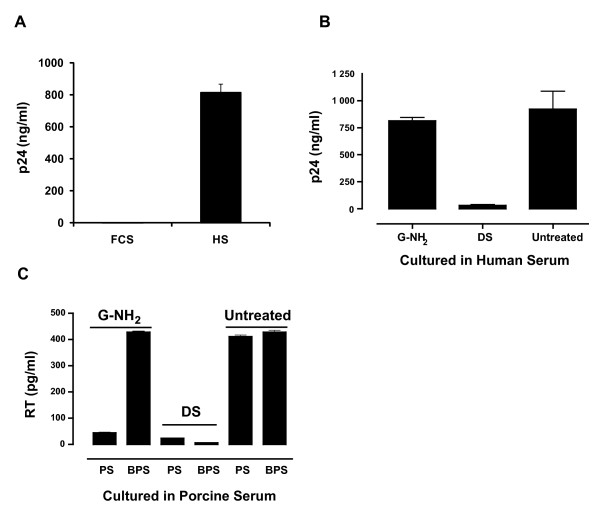
**Antiviral activity of G-NH_2 _and characterization of G-NH_2 _metabolite obtained after dialysis against FCS or PS**. (A) H9 cells (10^5^) were infected with the SF-2 strain of HIV-1 and cultured in medium containing 100 μM G-NH_2 _and either 10% fetal calf serum (FCS) or human serum (HS). Ten days post-infection, the level of p24-antigen in the culture supernatants was assayed with a p24-ELISA. (B) H9 cells infected with the SF-2 strain of HIV-1 were cultured in medium containing 10% human serum (HS) and either 100 μM G-NH_2 _or a dialysis solution (DS) of 1/10 dilution of 1 mM G-NH_2 _dialyzed against FCS. Untreated cultures without any addition of G-NH_2 _or DS served as controls. (C) Infected H9 cells were cultured in the presence of 10% boiled porcine serum (BPS) or non-boiled porcine serum (PS). The infected cultures were cultured with the addition of 100 μM G-NH_2_, DS or were left untreated. Virus production was assessed using an RT assay. Error bars indicate standard deviations from quadruple cultures.

The antiviral activity of G-NH_2 _was tested in HIV-1 infected H9 cells cultured in medium containing human (HS), porcine (PS) or fetal calf serum (FCS). When FCS was used, 100 μM G-NH_2 _repeatedly abolished HIV infectivity (Fig. 1A, FCS). Similar results were also obtained when infected cells were cultured in PS (data not shown). However, no antiviral activity was observed when the infected cells were cultured in HS (Fig. 1A, HS). An explanation for this could be that G-NH_2 _had to be converted by an enzyme present in FCS and PS, but not in HS, to acquire its antiviral activity. To test this hypothesis, 1 mM G-NH_2 _was dialyzed against FCS (pre-dialyzed against PBS five times to clear it from low molecular weight material) at 37°C over night. The dialysis solution (DS) obtained which contained the presumed G-NH_2 _metabolite, was then added to infected H9 cells cultured in medium containing HS (Fig. 1B, DS). Infected cell cultures to which 100 μM G-NH_2 _or no drug had been added served as controls. The results of a typical experiment are shown in Fig. 1B. G-NH_2 _showed no antiviral activity, however, infected cells cultured in human serum with DS at a 1/10 dilution, corresponding to ~100 μM of possible G-NH_2_-FCS product, showed virus replication that was completely inhibited (Fig. 1B, DS).

To further test if G-NH_2 _was converted to the active antiviral substance by an enzyme present in porcine or calf serum, HIV-1-infected H9 cells were cultured in medium containing normal PS or boiled PS (BPS). The cells were then treated with 100 μM G-NH_2_, with the DS at a 1/10 dilution or were left untreated. A typical experiment is depicted in Fig. 1C. Infected cells without any test compound and cultured in medium containing PS or BPS served as controls (Fig. 1C, Untreated). In contrast to what was observed in cells cultured with BPS and treated with DS, G-NH_2 _showed no antiviral activity in cells cultured with medium containing BPS (Fig. 1C). DS, however, repeatedly inhibited HIV replication regardless of the infected cells being cultured in the presence of PS or BPS (Fig. 1C, DS).

### HPLC analysis of the unknown metabolite of G-NH_2_

**Figure 2:**
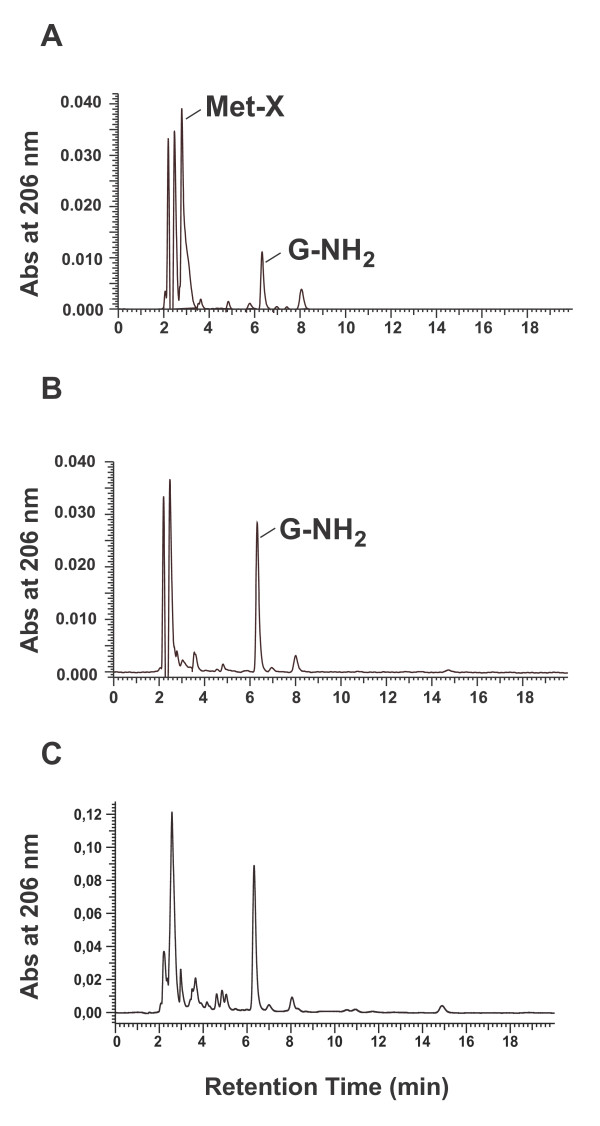
**HPLC analysis of G-NH_2 _and the G-NH_2_-derived metabolite Met-X**. HPLC chromatograms of dialysis solution (DS) after dialyzing 1 mM G-NH_2 _against porcine serum (PS) at 37°C (A) and at 4°C (B) or human serum at 37°C. The dialysis solutions were analyzed with a cationic ion-exchange column (Theoquest Hypersil SCX, Thermo), and the absorbance was measured at 206 nm.

DS derived from 1 mM G-NH_2 _dialyzed against pre-washed HS or PS was analyzed by HPLC using a cationic ion-exchange column. With G-NH_2 _dialyzed against PS at 37°C (Fig. 2A) but not at 4°C (Fig. 2B), a peak designated Met-X (retention time at 2.9 min) was always observed in addition to the G-NH_2 _peak (at 6.2 min). Dialysis of G-NH_2 _in HS gave no such change in the HPLC pattern (Fig. 2C). The unknown peak fraction obtained by dialysis of G-NH_2 _at 37°C was also isolated and tested for its antiviral activity (see below).

**Figure 3:**
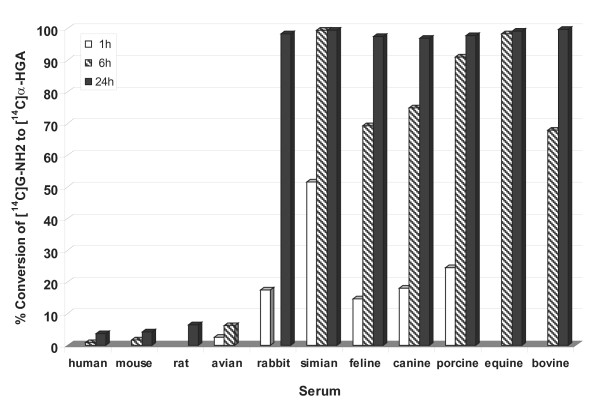
**Conversion of G-NH2 to Met-X in different sera**. ^14^C-labeled G-NH_2 _was incubated with sera from different species at different time points as indicated in the figure. Conversion to Met-X was analyzed by HPLC. Percent conversion to Met-X for respective sera is depicted.

Furthermore, we tested a number of different animal sera for their ability to convert ^14^C-G-NH_2 _to the antiviral metabolite X (Met-X). The conversion of G-NH_2 _to Met-X was detected by the migration pattern in HPLC. As shown in Figure 3, sera from human, rat, mouse, and bird did not convert G-NH_2 _but sera from rabbit, monkey, cat, dog, pig, horse and cow were successful in conversion.

### Identification of metabolite-X (Met-X) by NMR

**Figure 4:**
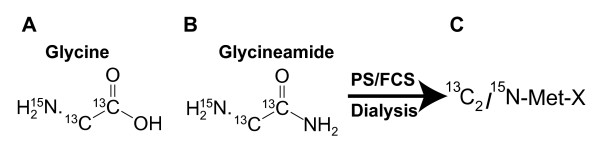
**Chemical structure and production of G-NH_2_-derived metabolite after dialysis against porcine serum**. The chemical structures of doubly labeled glycine with two ^13^C- and one ^15^N-isotopes (A) which was transformed into labeled glycineamide (B). The latter was dialyzed against porcine serum at 37°C, and the ^13^C_2 _^15^N-labeled product (C) here is referred to as Met-X. This compound was purified by HPLC and concentrated before being analyzed with NMR.

^13^C_2_/^15^N-labeled glycine (Fig. 4A) was transformed to ^13^C_2_/^15^N-labeled G-NH_2 _(Fig. 4B) by Fmoc peptide synthesis. In order to produce ^13^C_2_/^15^N-labeled Met-X (Fig. 4C), ^13^C_2_/^15^N-labeled G-NH_2 _(Fig. 4B) was dialyzed against PS or FCS as described above. The ^13^C^15^N-labeled Met-X was then purified by HPLC and the peak fraction containing labeled Met-X was concentrated by lyophilization before being analyzed by NMR.

Based on the NMR analysis (^1^H NMR, coupled ^1^H-^13^C NMR, and 2D ^1^H-^15^N HSQC NMR) one of the possible structures of the unknown compound Met-X was determined as α-hydroxy-glycineamide (α-HGA).

### Comparison of Met-X with α-HGA

**Figure 5:**
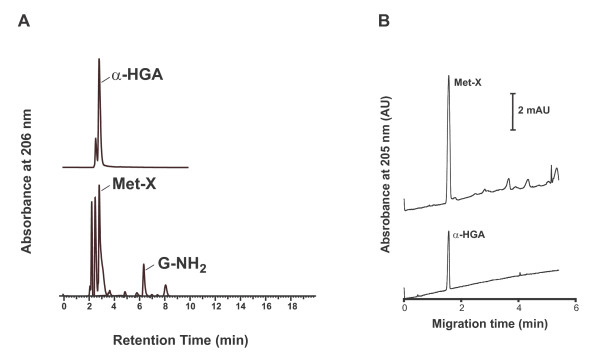
**Comparison of Met-X with α-HGA**. HPLC analysis of synthetically produced α-HGA and Met-X, the latter produced enzymatically after dialyzing 1 mM G-NH_2 _against PS at 37°C, is depicted in panel A, and capillary electrophoresis analysis of α-HGA and Met-X in panel B.

α-HGA was chemically synthesized and compared to Met-X. The HPLC chromatogram of α-HGA was identical to that of Met-X (Fig. 5A). Furthermore, the antiviral activity of the HPLC peak fraction identified as Met-X and α-HGA were tested in H9 cells infected with the HIV-1 SF-2 virus and in chronically infected ACH-2 cells. Both substances had similar antiviral activity (data not shown). The HPLC peak fractions identified as Met-X and α-HGA were also analyzed by capillary electrophoresis using bare silica capillaries under the same experimental conditions. The analysis of Met-X revealed some impurities which were well separated from the major peak containing Met-X (Fig. 5B). The pure α-HGA sample gave a symmetrical single peak, which had strikingly similar migration times to Met-X. The reproducibility was high (RSD = 1.14%; n = 4). Comparison of the UV spectra of the substances in separate experiments further revealed identical absorbance properties. Furthermore, the structural information gained by proton and carbon NMR analyses resulted in identical chemical shift values (data not shown).

Both α-HGA and Met-X treatment at concentrations corresponding to 10 μM resulted in similarly significant changes in virion core morphology (data not shown). Pleomorphic virus particles with distorted, irregular packing of aberrant core structures, partly devoid of dense core material, were seen. Virions having double core structures and occasionally viral cores bulging off from viral envelope were also observed.

### Anti-HIV activities of α-HGA and other related test compounds

**Figure 6:**
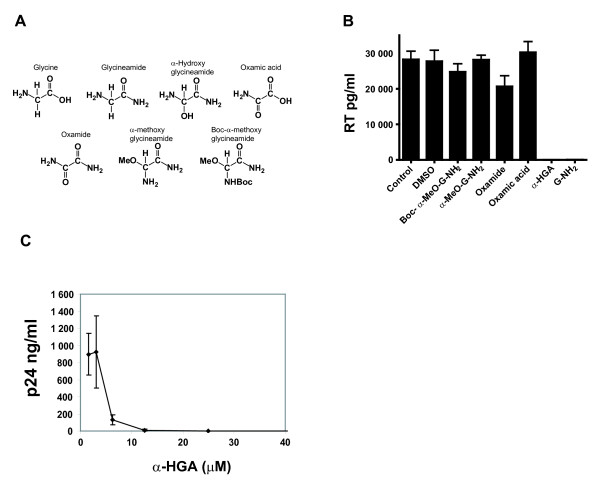
**Biological and antiviral comparison of α-HGA and some structurally related compounds**. Chemical structures of glycine, glycineamide (G-NH_2_), α-HGA, oxamic acid, oxamide, α-methoxy glycineamide and Boc-α-methoxy glycineamide (A). Antiviral activity of 100 μM of respective compound added to HIV-1 SF-2 infected H9 cells cultured in the presence of 10% fetal bovine serum (B). Dose response of the antiviral activity of synthetically produced α-HGA (C).

α-HGA and some other structurally related compounds (Fig. 6A) at drug concentrations of 100 μM were tested for a possible inhibitory effect on HIV-1 replication in infected H9 cells in the presence of FCS. As shown in Fig. 6B, both α-HGA and G-NH_2 _abolished HIV-1 replication. By contrast, oxamic acid, oxamide, α-methoxy glycineamide, and Boc-α-methoxy glycineamide did not show any significant effect on HIV-1 replication. The 50% inhibitory concentration (IC_50_) in HIV-1 SF-2 infected H9 cells ranged from 4 to 6 μM for both α-HGA and Met-X. A typical dose response curve for α-HGA is depicted in Fig. 6C.

## Discussion

In this study, we were able to identify, isolate and characterize a novel antiretroviral glycineamide (G-NH_2_)-derived metabolite (Met-X) obtained after incubation of G-NH_2 _in porcine (PS) or fetal calf (FCS) serum. Dialysis of G-NH_2 _against FCS at 4°C or boiled PS gave no Met-X, indicating that the enzyme responsible for converting G-NH_2 _to Met-X is temperature-dependent and heat-sensitive. Furthermore, unlike in FCS or in PS, G-NH_2 _could not be converted to Met-X when incubated in human serum at 37°C, suggesting that humans lack either the active enzyme or a necessary co-factor. Interestingly, humans seem to share this inability to convert G-NH_2 _with mice, rats and birds. However, other species such as non-human primates can readily convert G-NH_2 _to Met-X.

Here we characterized Met-X by NMR and this unknown compound was identified as α-hydroxy-glycineamide (α-HGA). In addition, with NMR, HPLC and capillary electrophoresis analysis of Met-X and the synthesized α-hydroxy-glycineamide the same chemical structure was determined. Therefore, it is very likely that these two compounds are identical chemical entities. The antiviral activity of the Met-X purified by cation exchange chromatography and identified as α-HGA by NMR was also confirmed both in H9 cells infected with the HIV-1 SF-2 virus and chronically infected ACH-2 cells. Consistent with previous reports on GPG-NH_2 _and G-NH_2_, the addition of Met-X or α-HGA to the culture medium of infected cells resulted in HIV-1 particles with aberrant core morphology.

The reduction in infectivity was not due to cytotoxicity, since neither Met-X nor α-HGA at concentrations up to 1,000 μM has any effect on the cell viability of PBMC or a number of other cell lines [27]. Furthermore, α-HGA had no mitogenic activity against human PBMCs at concentrations of up to 2,000 μM.

Two other compounds that inhibit or interfere with the HIV-1 capsid (p24/CA) maturation or assembly have previously been reported [20,21,28]. PA-457 [20,22], is a compound that binds to the proteolytic cleavage site of the p24 precursor (p25/CA-SP1) and thereby affects its maturation to p24. α-HGA does not affect the proteolytic processing of p25 [27]. The other compound reported by Tang *et al. *describes the binding of *N*-(3-chloro-4-methylphenyl)-*N'*-2-(5-[dimethylamino-methyl]-2-furyl)-methylsulfanyl-ethyl urea (CAP-1) to the N-terminal domain of p24 [21]. CAP-1 affects HIV-1 capsid cone formation but did not prevent virus release [21]. However, α-HGA, which is comparatively a small molecule, specifically affected HIV-1 CA assembly and cone formation, possibly by binding to the hinge region between the N- and C-terminal domains of p24 [27]. A 12-mer alpha-helical peptide (CAI) was also shown to interfere with p24 dimerization, but not with HIV-1 replication in cell culture due to the lack of cell penetration [28,29]. However, more recently a structure-based rational design was used to stabilize the alpha-helical structure of CAI and convert it to a cell-penetrating peptide (CPP) displaying antiviral activity [30].

## Conclusion

In this study, we have reported that G-NH_2 _by itself has no anti-viral activity but is converted to a small (molecular mass 90) anti-retroviral compound when incubated in some animal sera. The new compound was identified as α-HGA, which has an unusually simple structure and a novel mechanism of antiviral action. Thus, α-HGA could be a lead for new antiviral substances belonging to a new class of anti-HIV drugs, i.e. capsid assembly/maturation inhibitors.

## Methods

### Cells, media and reagents

Peripheral blood mononuclear cells (PBMC), H9 and ACH-2 cells were cultured in complete RPMI-1640, and HeLa-tat cells was cultured in complete DMEM medium supplemented with 10% serum and antibiotics. Porcine and human sera (PS and HS) (Biomeda), fetal calf serum (FCS; Invitrogen) oxamic acid and oxamide (Sigma) were used. Glycineamide (G-NH_2_) and α-hydroxy glycineamide (α-HGA; manufactured to order by Pharmatory Oy, Oulu, Finland) were kindly provided by Tripep AB, Stockholm, Sweden. ^13^C_2_/^15^N-labeled Fmoc-glycine (Isotech) was transformed to ^13^C_2_/^15^N-labeled G-NH_2 _by Fmoc peptide synthesis. ^13^C_2_/^15^N-labeled G-NH_2 _was dialyzed against PS or FCS to produce ^13^C_2_/^15^N-labeled metabolite of G-NH_2 _which will be referred to as Met-X (Fig. 2A, B and 2C).

### Inhibition of viral infectivity

HIV-1 stock of SF-2 from H9 cells was prepared as described previously [31], and 50% tissue culture infectious dose (TCID_50_) was determined. H9 cells were infected with SF-2 at 100 TCID_50 _by incubating for 2 hours at 37°C. The cells were then pelleted, washed and resuspended in complete RPMI medium containing HS or PS, and the test compound was added. Cells were cultured for 11 days, and the growth medium was changed seven days post-infection. The HIV-1 p24 antigen contents were assayed at day 7 and 11 post infection essentially as described elsewhere [32] (see below). For RT-assay, the manufacturer's procedure was followed (Cavidi Tech AB, Uppsala, Sweden).

### HPLC analysis and purification of Met-X

^13^C_2_/^15^N-labeled or unlabeled G-NH_2 _was enzymatically transformed to Met-X by dialysis against FCS or PS at 37°C. Dialysis was performed with 10 ml of serum in a dialysis tubing (5 kD MWCO) that was prewashed by dialyzing 5× against PBS under constant stirring. After 24 hours, the dialysis solution (DS) containing Met-X was analyzed by injecting it onto a 250 × 10 mm, 5 μm cationic ion-exchange column, Theoquest Hypersil SCX, (Thermo), with 90% 0.1 M KH_2_PO_4_pH 4.5/10% acetonitrile as mobile phase at isocratic flow. The absorbance was measured at 206 nm. Lyophilized ^13^C_2_/^15^N-labeled Met-X was also analyzed as above except that a mobile phase of 90% 2.5 mM formic acid pH 3/10% acetonitrile was used. All HPLC chromatograms were compared using retention time as an indicator. Once the structure of Met-X was indicated by NMR (see below) to be α-HGA, the HPLC properties of Met-X and chemically synthesized α-HGA were analyzed under the same conditions.

### Compound characterization by NMR spectroscopy

The HPLC peak fraction containing ^13^C_2_/^15^N-labeled Met-X was isolated, lyophilized, and analyzed with NMR. Due to the low natural abundance of ^13^C- and ^15^N-nuclei, a commercially available labeled glycine with two 99% ^13^C- and one 99% ^15^N-isotopes (Fig. 4A) was used as starting material. The labeled glycine was transformed into G-NH_2 _(Fig. 4B) which was dialyzed against PS or FCS to obtain labeled Met-X. The ^13^C/^15^N-labeled Met-X was purified by HPLC and concentrated by lyophilization before being analyzed with NMR. The samples were analyzed on a Bruker DPX 300 MHz, a Jeol Eclipse^+^500 MHz and Bruker DMX 600 MHz spectrometers.

### Comparison of Met-X with α-HGA by capillary electrophoresis

Capillary electrophoresis experiments were carried out at 20°C with a BioFocus 3000 system (Bio-Rad) which was equipped with a fast scanning UV-Vis detector. Fused silica tubing (50 and 365 μm inner and outer diameter, respectively) was purchased from MicroQuartz and cut to a length of 23 cm (with 18.5 cm effective length). Sodium phosphate buffer (0.05 M) at pH 7.4 was used as a background electrolyte. The polarity was set from positive to negative (with the detection point closer to the cathode). The capillary was flushed with the buffer for 1 minute before each run. The Met-X solution obtained from the dialysis procedure was diluted two fold in the buffer solution and filtered through a syringe disc filter (Ultra free-MC 5 000 NMWL, Millipore) prior to injection by pressure (3 psi·s). α-HGA was dissolved in the buffer at 10 mM concentration and injected by pressure (3 psi·s). The applied voltage was 10 kV in all experiments resulting in 50 μA current.

### ELISA

p24-ELISA of infected cell culture supernatants was performed essentially as described elsewhere [32]. Briefly, rabbit anti-p24 coated micro-well plates (MWP) were blocked with 3% BSA in PBS at 37°C for 30 minutes. Supernatants from infected cells were added to the plates, followed by incubated at 37°C for 1 hour. The MWPs were washed three times, and biotinylated anti-p24 antibody (1:1 500) was added. One hour after incubation, the MWPs were washed and incubated with HRP-conjugated streptavidine (1:2 000) for 30 minutes. Finally, the MWPs were washed and detected by adding the substrate o-Phenylenediamine Dihydrochloride (Sigma). Recombinant p24 at fixed concentrations was used as a standard. The plates were read in a Labsystems multiscan MS spectrometer. For RT-ELISA, the manufacturer's procedure was followed (Cavidi Tech).

### Anti-HIV activities of α-HGA and other related test compounds

The antiviral activity of α-HGA and some other structurally related compounds was tested in infected H9 cells in the presence of FCS at drug concentrations of 100 μM. H9 cells were infected as described above and cultured in medium containing oxamic acid, oxamide, α-methoxy glycineamide and Boc-α-methoxy glycineamide.

### Conversion of G-NH2 to α-HGA by different sera

The sera from different animal species were diluted 10-fold in 50 mM potassium phosphate buffer pH 8.0. To 100 μl (10% serum) were added 0.1 μCi [^14^C]G-NH_2 _(radiospecificity: 56 mCi/mmol), and the samples were incubated for 1, 6 or 24 hours at 37°C. At these time points, 200 μl cold methanol was added, and the samples were left on ice for another 15 minutes. After centrifugation at 15,000 rpm, the supernatants were subjected to HPLC analysis using a SCX-partisphere column (Whatman). The following gradient was used to separate G-NH_2 _and Met-X (α-HGA): 5 mM buffer B (5 mM NH_4_H_2_PO_4 _pH 3.5) (10 minutes); linear gradient to 83% buffer C (0.3 M NH_4_H_2_PO_4 _pH 3.5) (6 minutes); equilibration 83% buffer C (2 minutes); linear gradient to 100% buffer B (6 minutes); equilibration 100% buffer B (6 minutes). The retention times of G-NH_2 _and α-HGA were 12 and 2 minutes, respectively.

### Transmission electron microscopy (TEM)

H9 cells were infected with HIV-1 SF-2 at 100 TCID_50 _by incubating for 2 hours at 37°C. After seven days of incubation, medium containing Met-X or α-HGA was added. Cells were cultured for an additional four days, and progeny virus was analyzed by transmission electron microscopy (TEM). The HIV-1-infected H9 cells were fixed freshly upon embedding in epon, essentially as described before [24]. Sections were made approximately 60 nm thick to allow accommodation of the volume of the core structure parallel to the section plane. Duplicate samples were used and minimal beam dose technique was employed throughout. Evaluation of morphology was done with series of electron micrographs to depict different categories of virus morphology. Similar results were also obtained with chronically infected ACH-2 cells induced to replicate HIV-1.

## Competing interests

Author AV is a shareholder and a director of the board of Tripep AB, and author ML is an employee of Tripep AB.

## Authors' contributions

SH performed electron microscopy, ÁV capillary electrophoresis, and ML HPLC analysis. MH together with WT and IR performed NMR analysis. JB performed the experiments with different animal sera. SA performed all other experiments in the study and wrote the manuscript with AV. AV is the principal investigator and conceived of the study. All authors read and approved the manuscript.
